# Updated Dosing Instructions for Immune Globulin (Human) GamaSTAN S/D for Hepatitis A Virus Prophylaxis

**DOI:** 10.15585/mmwr.mm6636a5

**Published:** 2017-09-15

**Authors:** Noele P. Nelson

**Affiliations:** 1 Division of Viral Hepatitis, National Center for HIV/AIDS, Viral Hepatitis, STD, and TB Prevention, CDC.

GamaSTAN S/D (Grifols Therapeutics, Inc., Research Triangle Park, North Carolina) is a sterile, preservative-free solution of immune globulin (IG) for intramuscular administration and is used for prophylaxis against disease caused by infection with hepatitis A, measles, varicella, and rubella viruses (*1*). GamaSTAN S/D is the only IG product approved by the Food and Drug Administration for hepatitis A virus (HAV) prophylaxis. In July 2017, GamaSTAN S/D prescribing information was updated with changes to the dosing instructions for hepatitis A preexposure and postexposure prophylaxis indications. These changes were made because of concerns about decreased HAV immunoglobulin G antibody (anti-HAV IgG) potency, likely resulting from decreasing prevalence of previous HAV infection among plasma donors, leading to declining anti-HAV antibody levels in donor plasma (*2*). No changes in dosing instructions were made for measles, varicella, or rubella preexposure or postexposure prophylaxis.

Following are the updated recommended doses of GamaSTAN S/D for hepatitis A preexposure and postexposure prophylaxis (*2*).

## Preexposure Prophylaxis in Persons Who Plan to Travel in Areas with High or Intermediate Hepatitis A Endemicity

The recommended dosages of GamaSTAN S/D, which vary according to planned duration of travel are as follows (Table):

**TABLE:** Indications and updated dosage recommendations for GamaSTAN S/D human immune globulin for preexposure and postexposure prophylaxis against hepatitis A infection

Indication	Updated dosage recommendation
**Preexposure prophylaxis**	
Up to 1 month of travel	0.1 mL/kg
Up to 2 months of travel	0.2 mL/kg
2 months of travel or longer	0.2 mL/kg (repeat every 2 months)
**Postexposure prophylaxis**	0.1 mL/kg

• Up to 1 month: 0.1 mL/kg

• Up to 2 months: 0.2 mL/kg

• 2 months or longer: repeat dose of 0.2 mL/kg every 2 months.

## Postexposure Prophylaxis of Household and Institutional Hepatitis A Case Contacts

The recommended dosage of GamaSTAN S/D is 0.1 mL/kg (Table). There is no maximum dosage of GamaSTAN S/D for hepatitis A prophylaxis (*1*).

The effect of IG preparations on the response to certain live-virus vaccines is unknown, but antibodies in GamaSTAN S/D might interfere with live-virus vaccines such as measles, mumps, and rubella (MMR) vaccine and varicella vaccine (*1*,*3*). The recommendations for the timing of administration of GamaSTAN S/D with live-virus vaccines has not changed (*1*,*3*). The Advisory Committee on Immunization Practices (ACIP) recommends that MMR and varicella vaccines should be administered at least 2 weeks before or at least 3 months after the administration of IG preparations (*1*). If an IG preparation must be administered less than 2 weeks after the administration of MMR or varicella vaccine, the patient should be revaccinated no sooner than 3 months after receipt of the IG preparation.

The absolute lower limit of anti-HAV IgG required to prevent HAV infection has not been defined; however, 10 mIU/mL is considered to be the minimum protective level for HAV prophylaxis (*1*,*4*). The minimum anti-HAV IgG potency specified by the European Pharmacopoeia for intramuscular IG preparations indicated for HAV prophylaxis is >100 IU/mL (*5*). A recent study showed that only two of nine tested lots of commercially available IG preparations manufactured in the United States, Europe, and Asia had anti-HAV IgG potency of 100 IU/mL (*6*). In addition, anti-HAV IgG decay models indicate that only five of nine lots of IG dosed at 0.02 mL/kg achieved postabsorption plasma anti-HAV IgG levels above the minimum protective level of 10 mIU/mL (*6*). The decay model also showed that none of the tested IG lots maintained the proposed minimal protective anti-HAV IgG level of 10 mIU/mL for 3 months (*6*).

Indications for the use of IG are based on ACIP recommendations published in 2007 for prevention of hepatitis A infection after exposure to HAV and in international travelers (*7*).

## Preexposure Prophylaxis for International Travel

Hepatitis A vaccine at the age-appropriate dose is preferred to IG. For travel that will begin in ≤2 weeks to countries with high or intermediate hepatitis A endemicity, older adults, immunocompromised persons, and persons with chronic liver disease or other chronic medical conditions may receive IG simultaneously with hepatitis A vaccine at a separate anatomic injection site. Travelers who elect not to receive hepatitis A vaccine, who are aged <12 months, or who are allergic to a component of hepatitis A vaccine should receive a single dose of IG before travel (*7*).

## Postexposure Prophylaxis

IG should be used for children aged <12 months, immunocompromised persons, persons who have chronic liver disease, and persons for whom vaccine is contraindicated. IG is also preferred over hepatitis A vaccine for persons aged >40 years; however, vaccine may be used if IG cannot be obtained (*7*).
